# Comparative Analysis of Mitochondrial Genomes in Two Subspecies of the Sunwatcher Toad-Headed Agama (*Phrynocephalus helioscopus*): Prevalent Intraspecific Gene Rearrangements in *Phrynocephalus*

**DOI:** 10.3390/genes13020203

**Published:** 2022-01-23

**Authors:** Na Wu, Jinlong Liu, Song Wang, Xianguang Guo

**Affiliations:** 1 Chengdu Institute of Biology, Chinese Academy of Sciences, Chengdu 610041, China; wuna@cib.ac.cn (N.W.); liujl@cib.ac.cn (J.L.); wangsong0311@outlook.com (S.W.); 2 University of Chinese Academy of Sciences, Beijing 100049, China; 3 College of Life Sciences and Technology, Inner Mongolia Normal University, Hohhot 010022, China

**Keywords:** mitochondrial genome, next-generation sequencing, gene rearrangement, *Phrynocephalus*, phylogenetic analysis, divergence time, tRNA duplication

## Abstract

Intraspecific rearrangements of mitochondrial genomes are rarely reported in reptiles, even in vertebrates. The sunwatcher toad-headed agama, *Phryncoephalus helioscopus*, can serve as an excellent model for investigating the dynamic mitogenome structure at intraspecific level. To date, seven subspecies of *P. helioscopus* are well recognized, but little is known about the mitogenomic evolution among different subspecies. In this study, complete mitogenomes of subspecies *P. helioscopus varius* II and *P. helioscopus cameranoi* were determined by next-generation sequencing, and another *P. helioscopus varius* I retrieved from GenBank was compiled for comparative analysis. The nucleotide composition and the codon usage are similar to those previously published from toad-headed agamas. *P. helioscopus varius* II and *P. helioscopus cameranoi* have 23 tRNA genes, including standard 22 tRNA genes and one extra *tRNA-Phe* (*tRNA-Phe* duplication). Gene order and phylogenetic analyses in the genus *Phrynocephalus* support prevalent intraspecific gene rearrangement in *P. helioscopus* and other congener species including *P. erythrurus*, *P. vlangalii*, and *P. forsythii*. Six different mitochondrial gene arrangements are observed in *Phrynocephalus*. Overall, the occurrence of rearrangements may result from multiple independent structural dynamic events. The split of the two subspecies in *P. helioscopus* was dated at approximately 2.34 million years ago (Ma). Two types of gene rearrangements are found in the three mitogenomes of *P. helioscopus*, and this intraspecific rearrangement phenomenon can be explained by the tandem duplication/random loss (TDRL) model. Post duplication, the alternative loss types can occur in 0.23–0.72 Ma, suggesting that the duplication and fixation of these rearrangements can occur quite quickly. These findings highlight the need for more mitogenomes at the population level in order to better understand the potentially rampant intraspecific mitogenomic reorganization in *Phrynocephalus*.

## 1. Introduction

It is well known that the structure of the vertebrate mitochondrial genome (mitogenome) is compact and relatively conservative, containing 37 genes (13 protein-coding genes, two ribosomal RNA genes and 22 transfer RNA genes) as well as a control region (CR; also called D-loop) [1,2,3]. The advancement of sequencing technology including next-generation sequencing has facilitated rapid availability of mitogenome of animals from various groups [4,5]. Accordingly, gene rearrangement phenomena have been more and more discovered in various vertebrate lineages [6,7,8,9,10,11,12,13,14]. Nevertheless, intraspecific gene rearrangements are not commonly found through comparison of all mitochondrial DNA records of the squamate reptiles [15]. Two cases occur in the squamates, one in asexual geckos with multiple origins of duplication [16] and another in an amphisbaenian (worm lizard) with alternative loss types varying among populations [17]. Thus, investigations at intraspecific level may be necessary to understand the evolution of mitochondrial gene rearrangements.

The tandem duplication followed by the random loss (TDRL) is the most frequently invoked model to understand the diversity of gene rearrangements in vertebrate mitogenomes [7]. According to the TDRL model, random deletion of redundant duplicated genes results in novel gene orders [6,7]. The agamid genus *Phrynocephalus*, known as toad-headed agama or toad-headed lizard, is one of the most speciose reptile genera in arid central Asia. As of 31 July 2021, a total of 23 mitogenomes representing 14 species in the genus *Phrynocephalus* have been released in GenBank. Several cases of gene rearrangements in the mitogenomes of the genus *Phrynocephalus* have been reported. Liu et al. [18] summarized five rearrangement types (V, VI, VII, VIII, and IX) of mitogenomes among 12 species in *Phrynocephalus*. The mitogenome structures of *P. przewalskii*, *P. versicolor*, and *P. frontalis* have been reported to have tRNA genes duplication [19,20,21]. However, as for the mitogenomics of *Phrynocephalus*, exploration of the potential mechanism of gene rearrangements is still lacking.

The sunwatcher toad-headed agama, *P. helioscopus* (Pallas, 1771)*,* is one of the most taxonomically complex taxa within the toad-headed agamas [22,23,24]. As one of the most typical and widespread desert lizards in the arid central Asia, *P. helioscopus* occurs in a variety of habitats from sand dunes and gravel deserts to steppe deserts [23,24,25,26,27,28]. It is now widely recognized that *P. helioscopus* constitutes a complex assembly of more or less separated populations and subspecies. In combination with mitochondrial DNA and morphological characters, *P. helioscopus* was classified into seven subspecies [22,23], i.e., *P. helioscopus helioscopus*, *P. helioscopus cameranoi* Bedriaga, 1907, *P. helioscopus meridionalis* Dunayev, Soloveyeva, and Poyarkov, 2012, *P. helioscopus saidalievi* Sattorov, 1981, *P. helioscopus sergeevi* Dunayev, Soloveyeva, and Poyarkov, 2012, *P. helioscopus turcomanus* Dunayev, Soloveyeva, and Poyarkov, 2012, and *P. helioscopus varius* Eichwald, 1831. Among these subspecies, two are permeated in Northwest China, i.e., *P. helioscopus varius* from eastern Kazakhstan through Xinjiang Uygur Autonomous Region, China to western Mongolia, and *P. helioscopus cameranoi* from east Kazakhstan to the Chinese part of the Ily River Valley. Thus, *P. helioscopus* can serve as an excellent model for investigating the dynamic mitogenome structure at intraspecific level.

As such, the starting point of this study is to investigate whether there is intraspecific rearrangement in the mitogenomes of *P. helioscopus*. If so, we will further explore the underlying arrangement process. We used next-generation sequencing to produce complete mitognomes of two subspecies, i.e., *P. helioscopus varius* and *P. helioscopus cameranoi*. Specifically, we aimed to (i) document the conservation or variation between the two subspecies’ mitogenomes in genome size, genomic features, and gene content; (ii) determine the structure of the rearranged region at subspecies level, and infer the steps resulting in observed gene rearrangements; and (iii) estimate the time of intraspecific gene rearrangements by Bayesian phylogenetic dating. The findings will provide clues to understand the multiple independent structural dynamic events in the mitogenomic evolution of the toad-headed agamas.

## 2. Materials and Methods

### 2.1. Sampling and DNA Extraction

**Figure 1:**
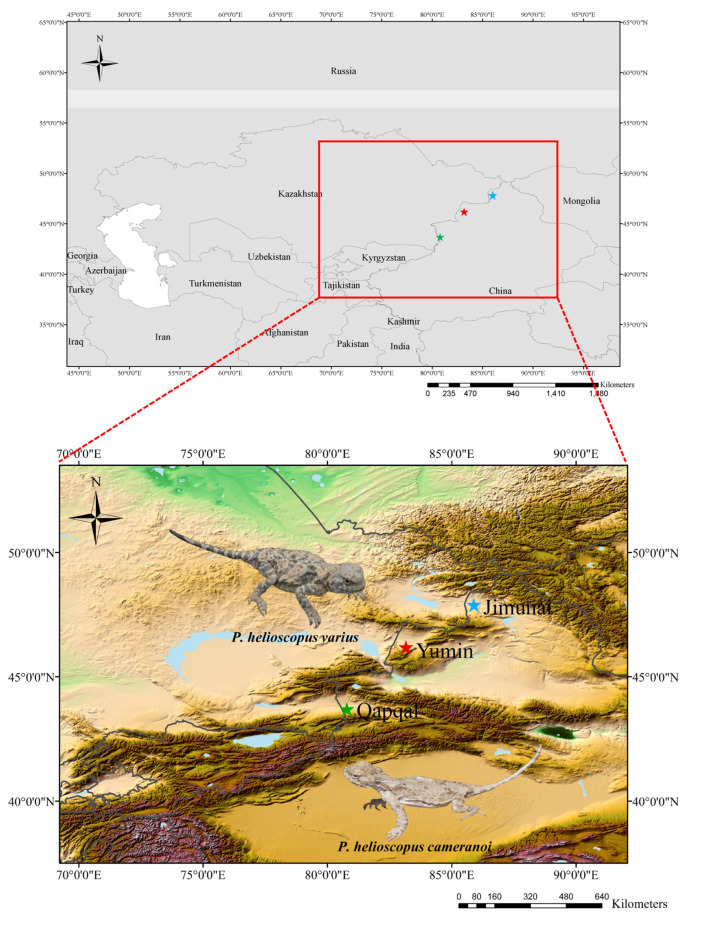
Sampling sites for the specimens of *P. helioscopus* in Xinjiang Uygur Autonomous Region, China. 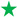, *P. helioscopus cameranoi*, from Qapqal (Ily River Valley; sequenced in this study). 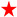, *P. helioscopus varius* II, from Yumin (Tacheng Prefecture, north of the Tianshan; sequenced in this study). 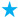, *P. helioscopus varius* I, from Jimunai [29] (Altay Prefecture, north of the Tianshan; retrieved from GenBank under accession number KM093858). Photos were taken by Jinlong Liu.

The specimen of *P. helioscopus varius* was captured from Yumin County (43.69313° N; 80.78233° E), Tacheng Prefecture, Xinjiang Uygur Autonomous Region, China (voucher no. GXG345, called *P. helioscopus varius* II in this paper) on 1 July 2017, and the specimen of *P. helioscopus cameranoi* (voucher no. Guo6379) was captured from Qapqal Xibe Autonomous County (46.18425° N; 83.16024° E), Yili Kazak Autonomous Prefecture, Xinjiang Uygur Autonomous Region, China on 14 July 2019 (Figure 1). Specimens were identified by their morphological characteristics [23]. A liver sample was dissected from the euthanized lizard with an overdose of sodium pentobarbital delivered by intraperitoneal injection. Both the liver samples and voucher specimens were fixed in 95% ethanol and deposited in the Chengdu Institute of Biology (CIB), Chinese Academy of Sciences. All animal procedures were approved by the Animal Care and Use Committee of CIB (Permit Number: CIB-20121220A).

Total genomic DNA was extracted from the liver tissue. The integrity of DNA samples was measured using 1% agarose gel electrophoresis and by the HiPure Universal DNA Kit (Magen Biotech, Guangzhou, China) following the manufacturer’s protocol for UV spectroscopy. DNA concentration and purity were measured by NanoDrop 2000 spectrophotometer (Thermo Scientific, Wilmington, NC, USA) and Qubit 2.0 Flurometer (Invitrogen, Carlsbad, CA, USA). Qualified DNA samples were stored at −20 °C for the next experiment.

### 2.2. Library Construction and High-Troughput Sequencing

High-quality DNA samples were randomly fragmented with a size of 350 bp for paired-end sequencing, and DNA libraries were constructed according to the standard procedure of Illumina DNA library construction in the Genepioneer Biotechnologies Co. Ltd. (Nanjing, China). The library (inserted size of 350 bp) was constructed using the VAHTS^®^ Universal DNA Library Prep Kit. The quality of the library was control by qPCR method and Agilent 2100 Bioanalyzer (Agilent, Santa Clara, CA, USA). The quality-qualified DNA library was run on an Illumina NovaSeq (Illumina, , San Diego, CA, USA) with paired-end reads of 150 bp in the Genepioneer Biotechnologies Co. Ltd. (Nanjing, China).

### 2.3. Sequence Assembly, Annotation, and Analysis

The raw data obtained by Illumina NovaSeq sequencing were filtered to get high-quality sequences with fastp v.0.20.0 [30], by trimming adapters and primers, filtering reads with phred quality < Q5 and N base number > 5. The obtained high-quality fragments were aligned with *P. helioscopus* mtiogenome in GenBank (accession no. KM093858) to remove sequence repeats and inaccurate sequencing, and then assembled by SPAdes v.3.10.1 [31] to obtain the complete circular mitogenome. The preliminary annotations of mitochondrial genome were performed in the MITOS Web Server [32,33]. The protein-coding genes and ribosomal genes were determined by alignment with the reported mitochondrial genomes of the congeners based on the methods of Blastn [34]. Twenty-two transfer RNAs (tRNAs) genes and their potential secondary structures were verified using tRNA scan-SE software [35,36].

Mitochondrial gene structure maps were drawn using the online tool OGDRAW v.1.3.1 [37,38]. In order to conduct comparative analysis of the mitochondrial genomes of two subspecies of *P. helioscopus*, another published sequence of *P. helioscopus* (accession no. KM093858, from Jimunai County, Altay Prefecture, Xinjiang Uygur Autonomous Region, called *P. helioscopus varius* I in this paper) was also included. The nucleotide composition, codon distribution, and relative synonymous codon usage (RSCU) of PCGs values were analyzed with MEGA v.7.0 [39]. Composition skew values were calculated using the formulae: AT-skew = (A% − T%) / (A% + T%); GC-skew = (G% − C%) / (G% + C%) [40].

### 2.4. Phylogenetic Analyses

Phylogenetic analyses were performed based on 27 available mitogenomes, including *P. helioscopus cameranoi* and *P. helioscopus varius* II, 23 published sequences of *Phrynocephalus*, and two outgroups (Table S1). *Pseudotrapelus sinaitus* and *Xenagama taylori* were selected as outgroup taxa based on current understanding of the phylogenetic relationship among agamid lizards and the available data in GeneBank [41,42,43,44]. The 13 PCGs were used with a total length of 11,028 bp in our analyses. The 13 PCGs were aligned in MAFFT v.7.313 [45] with the default parameters and were concatenated by the plug-in concatenate sequence option in PhyloSuite v.1.2.2 [46]. The optimal partitioning scheme for nucleotide substitution models for each gene were determined by PartitionFinder v.2.0 [47] implemented in the PhyloSuite under a greedy search algorithm with linked branch lengths based on the Bayesian information criterion (BIC). The best-fit substitution models and partitioning schemes for each gene are listed in Table S2.

Phylogenetic trees were reconstructed using Maximum Likelihood (ML) and Bayesian Inference (BI) methods. The ML analyses were implemented in IQ-TREE v.1.6.8 [48] under the models selected for each identified partition. Branch support analyses were conducted under 5000 ultrafast-bootstrap replicates (UFBoot) [49]. Nodes with UFBoot ≥ 95 were considered to be well-supported [49]. Partitioned Bayesian analyses were performed in MrBayes v.3.2.6 [50] based on the optimal model of each partition. Two independent runs of four Monte Carlo Markov chains (MCMCs) were simultaneously run for two million generations, with sampling conducted every 100 generations. The convergence of the independent runs was indicated by a standard deviation of split frequencies < 0.01, and the effective sample size (ESS) > 200 calculated in Tracer v.1.7.1 [51]. A 50% majority-rule consensus tree and posterior probability (PP) of clades were assessed from the trees after the initial 25% trees of each MCMC run were discarded as burn-in. The phylogenetic trees were viewed and edited with FigTree v.1.4.4 [52] and PowerPoint.

### 2.5. Molecular Dating Analyses

Given that different species usually had different molecular substitution rates, a relaxed molecular clock method was used within the program BEAST v.1.8.4 [53] to estimate divergence time among *Phrynocephalus* species, specifically the three *P. helioscopus* individuals. This was performed using a concatenated dataset of 13 protein-coding genes. Best partition schemes and corresponding models were inferred using PartitionFinder v.2.0 [47] under the Bayesian Information Criterion (BIC) (Table S2). All mitochondrial data sets were run with linked trees, clock models, and unlinked sites. Due to a lack of reliable fossil evidence, two calibration points described in a previous study were employed in the divergence time estimation [21]. The first calibration point, C1, representing the divergence time of genus *Phrynocephalus* was set at 9.78 million years ago (Ma; 95% credible interval: 6.49–13.07 Ma). The second calibration point, C2, based on the result of Jin and Brown [21] on the MRCA of viviparous species, was dated to 5.04 Ma (95% credible interval: 2.57–7.51 Ma). Two independent runs of 20 million generations were performed with birth-death speciation tree prior and a sampling frequency of 2000 generations. Convergence for all model parameters was assessed by examining trace plots and histograms in Tracer v.1.7.1 [51] after obtaining an effective sample size (ESS) > 200. Runs were combined using LogCombiner (discarding 25% of the initial runs), and maximum credibility trees with divergence time means and 95% highest posterior densities (HPDs) were produced using TreeAnnotator (both part of the BEAST package). 

## 3. Results and Discussion

### 3.1. Genome Organization and Composition

**Figure 2:**
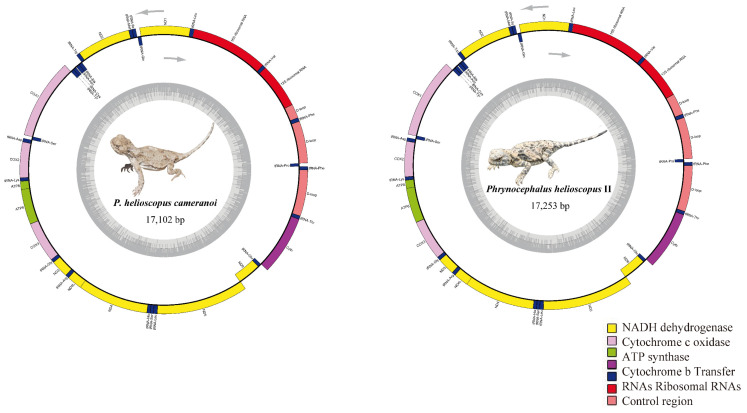
Mitochondrial genome map of *P. helioscopus cameranoi* and *P. helioscopus varius* II. Genes encoded on the heavy or light strand are indicated on the outside or inside of the circular mitogenome map, respectively, showing the direction of transcription. The tRNAs are denoted by color and labeled according to the three-letter amino acid codes. Lizard photos were taken by Jinlong Liu.

The complete mitogenomes of *P. helioscopus varius* II and *P. helioscopus cameranoi*, 17,253 bp and 17,102 bp in size, respectively, were circular DNA molecules (Figure 2; Table S3b,c). Notably, by re-annotating the mitogenome of *P. helioscopus varius* I (accession number KM093858), we found that the originally annotated *tRNA-Pro* did belong to part of the CR. Meanwhile, the alignment of KM093858 only resulted in a 16 bp length of *tRNA-Pro* gene (the position can be determined), so KM093858 was recognized as a nearly complete mitogenome, with a length of 16,249 bp (Table S3a). The three mitogenomes have genetic content typical of most vertebrate mitogenomes [2,54], including 13 protein coding genes (PCGs) and two rRNA genes (*12S* and *16S*). In addition to the typical 22 tRNA genes commonly found in the mitogenomes of both *P. helioscopus varius* II and *P. helioscopus cameranoi*, an additional *tRNA-Phe* was identified in them. However, only 21 tRNA genes were recognized in *P. helioscopus varius* I—an incomplete *tRNA-Pro* (only 16 bp) and no *tRNA-Phe* duplication (Table S3a). Most of the mtDNA genes are encoded on the H-strand, except for *ND6* and eight tRNA genes (*tRNA-Pro*, *tRNA-Gln*, *tRNA-Ala*, *tRNA-Asn*, *tRNA-Cys*, *tRNA-Tyr*, *tRNA-Ser* (UCN), and *tRNA-Glu*), which are encoded on the L-strand (Table S3). Gene overlaps were observed at eight locations, and the longest overlap involved 13 bp between *ND5* and *ND6*. Overall, nine intergenic/non-coding regions are present, ranging in length from 1 to 16 bp.

Nucleotide composition analysis indicated that the three mitogenomes displayed a significant bias toward adenine (A)/thymine (T), with overall A + T content of 62.3% in *P. helioscopus varius* I, 62.4% in *P. helioscopus varius* II, and 62.3% in *P. helioscopus cameranoi* (Table S4). This base pattern is congruent with most previously reported mitogenomes in *Phrynocephalus* [55,56,57,58,59]. The PCGs, rRNAs, and tRNAs were also A/T-biased in nucleotide composition. The A + T content composition in regions of *P. helioscopus varius* I was 62.7% (PCGs), 61.3% (tRNAs), and 59.7% (rRNAs); 62.8% (PCGs), 61.4% (tRNAs), and 59.7% (rRNAs) in *P. helioscopus varius* II; and 62.9% (PCGs), 61.4% (tRNAs), and 59.3% (rRNAs) in *P. helioscopus cameranoi*. In addition, the skew metrics of the three mitogenomes showed AT-skew value of each region (except for the CRs) was positive (0.055 to 0.275), which implied that A had more content than T. In contrast, except for tRNA genes region of *P. helioscopus varius* II and *P. helioscopus cameranoi*, the CG-skew value of each region was negative (−0.002 to −0.354), indicating a higher content of C than G (see Table S4 for details).

### 3.2. Protein-Coding Genes (PCGs) and Codon Usage

In the mitogenome of *P. helioscopus varius* I, *P. helioscopus varius* II, and *P. helioscopus cameranoi*, the total length of PCGs was 11,249 bp, 11,252 bp, and 11,246 bp, accounting for 69.2%, 65.2%, and 65.8% of the whole mitogenome, respectively (Table S4). The initiation codon of PCGs of the three mitogenomes are mostly ATN (ATG, ATT, ATA, and ATC), except for *ATP8*, *ND4L*, and *ND6* genes, which have GTG as the initiation codon (Table S3). Most of the PCGs terminate with TAA or TAG stop codons, except that termination codons of *ND2*, *COX1*, and *ND6* genes are AGA, AGA, and AGG, respectively. In addition, five mitochondrial genes (*COX2*, *ATP6*, *COX3*, *ND3*, *Cytb*) in *P. helioscopus* are terminated with an incomplete stop codon T--.

**Figure 3:**
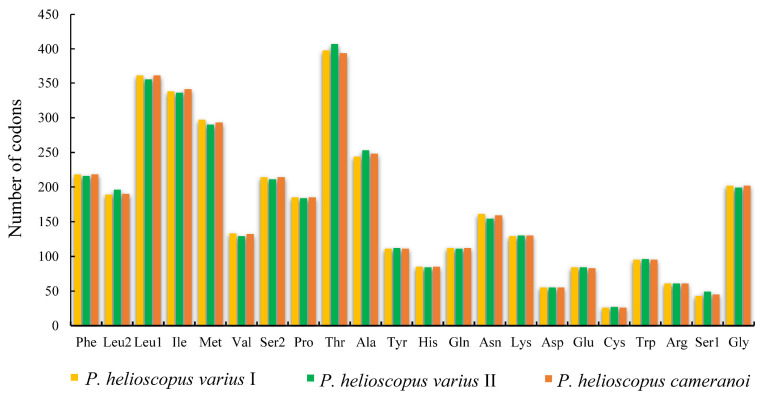
Amino acid frequency in the mitogenomes of *P. helioscopus varius* I, *P. helioscopus varius* II, and *P. helioscopus cameranoi*. Numbers on the *Y*-axis refer to the total number of the codons and codon families are provided on the *X*-axis.

**Figure 4:**
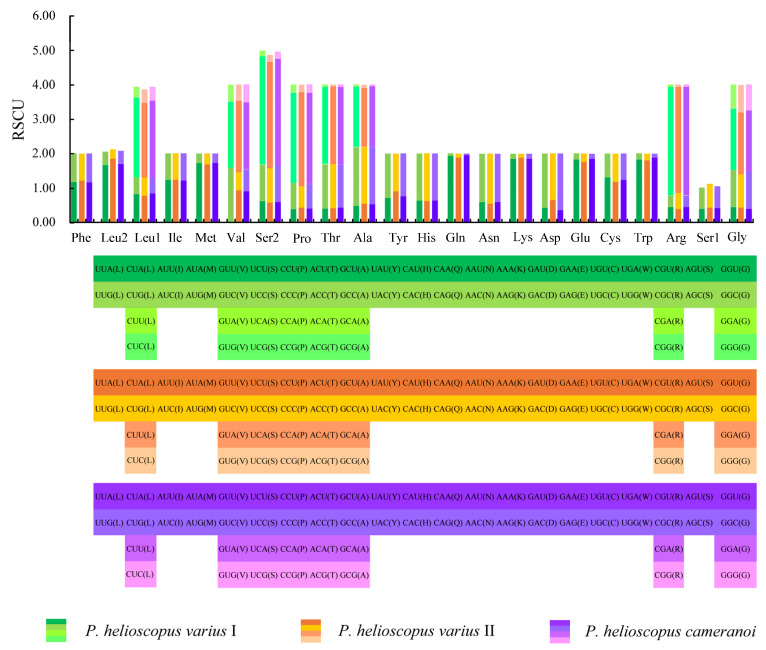
The relative synonymous codon usage (RSCU) in the mitogenomes of *P. helioscopus varius* I, *P. helioscopus varius* II, and *P. helioscopus cameranoi*. The codons are shown on the *X*-axis and the RSCU values are shown on the *Y*-axis.

The total number of codons in PCGs is 3748 in *P. helioscopus varius* I, 3749 in *P. helioscopus varius* II, and 3747 in *P. helioscopus cameranoi*. Comparative analysis showed that codon usage patterns of three mitogenomes were highly conserved. The most frequently encoded amino acid by codons are Threonine (Thr), Leucine-CUN (Leu1), and Isoleucine (Ile), while those encoding Cys, Ser-AGY (Ser1), and Asp are rare (Figure 3; Table S5). Meanwhile, the relative synonymous codon usage (RSCU) values of three mitogenomes were calculated and are summarized in Figure 4 and Table S5. Comparative analysis found that the relative synonymous codon usages (RSCU) of the three *P. helioscopus* mitochondrial PCGs were basically similar. Among them, UCA-Ser2, CGA-Arg, and CCA-Pro were the most frequently used codons.

### 3.3. Transfer RNAs and Ribosomal RNAs

The typical 22 tRNA genes and the additional *tRNA-Phe* were detected in *P. helioscopus varius* II and *P. helioscopus cameranoi* (Figure S1, Table S3). In contrast, only 21 tRNA genes were observed in *P. helioscopus varius* I, with an incomplete *tRNA-Pro* and without *tRNA-Phe* duplication. The total length of tRNAs of the three mitogenomes is 1428 bp in *P. helioscopus varius* I, 1542 bp in *P. helioscopus varius* II, 1546 bp in *P. helioscopus cameranoi*, respectively. Most of the tRNAs are able to fold into a typical cloverleaf secondary structure, but with a few exceptions. Overall, *tRNA-Ser1^AGY^* and *tRNA-Cys* lack a dihydrouridine (DHU) stem-loop structure, *tRNA-Thr* lacked TΨC loop (Figure S1, Table S3). Specifically, in *P. helioscopus varius* I, *tRNA-Pro* fails to form a cloverleaf secondary structure due to its incompleteness.

Both *12S rRNA* and *16S rRNA* genes are encoded from the H-strand in the three *P. helioscopus* mitogenomes (Table S3). The two rRNAs of *P. helioscopus* are located between the control region (CR) and *tRNA-Leu (UUR)* and separated by *tRNA-Val.* The length of *12S rRNA* is 865 bp for *P. helioscopus varius* I, 867 bp for *P. helioscopus varius* II, and 868 bp for *P. helioscopus cameranoi*. The length of *16S rRNA* is 1484 bp for *P. helioscopus varius* I, 1485 bp for *P. helioscopus varius* II, and 1484 bp for *P. helioscopus cameranoi* (Table S3).

### 3.4. Control Region

The control region is the largest functional non-coding entity in the mitogenome and is heavily biased to A+T nucleotides. The CRs of *Phrynocephalus* species vary in number and location within the genome due to duplications and gene rearrangements. In the three mitogenomes of *P. helioscopus*, three CRs are observed in the *P. helioscopus varius* II and *P. helioscopus cameranoi*, two in *P. helioscopus varius* I. The CRI is located between *tRNA-Thr* and *tRNA-Phe* in the three *P. helioscopus* mitogenomes with a length of 892 bp, 893 bp, and 889 bp, respectively. This is similar to the typical position of the CR of vertebrate mitogenomes known to date. The CRII of *P. helioscopus varius* II and *P. helioscopus cameranoi* are located between *tRNA-Pro* and *tRNA-Phe* with a length of 804 bp and 806 bp, respectively; while the CRII of *P. helioscopus varius* I is located between *tRNA-Phe* and *12S rRNA* with a length of 334 bp. The CRIII of *P. helioscopus varius* II and *P. helioscopus cameranoi* are located between *tRNA-Phe* and *12S rRNA* with a length of 417 bp and 272 bp, respectively. It is notable that the sequences of the CRI of the three mitogenomes are highly similar, and the sequences of CRII and CRI are also very similar (except for *P. helioscopus varius* I), while the CRII of *P. helioscopus varius* I is similar to the CRIII of *P. helioscopus varius* II and *P. helioscopus cameranoi*. In addition, we observed a conserved motif (CSB1: CTTTTCATGCTCAGTAGACATA) in the CRI of the three mitogenomes and in the CRII of *P. helioscopus varius* II and *P. helioscopus cameranoi*.

### 3.5. Gene Rearrangement

Mitogenomes of vertebrates are generally compact and relatively conserved [2]. With the massive increase of the whole mitogenome data of vertebrates, the gene rearrangement phenomena were commonly encountered in mitogenomes of vertebrates [6,9,60,61,62]. By investigating the mitogenomes of 27 Agamidae species, Liu et al. [18] summarized nine types of mitochondrial gene rearrangement (Type I−IX) and listed the rearrangement patterns of the mitogenomes of 12 *Phrynocephalus* species. Liu and colleagues provided fundamental information for us to assess the mitochondrial gene rearrangement phenomena in *Phrynocephalus.*

**Figure 5:**
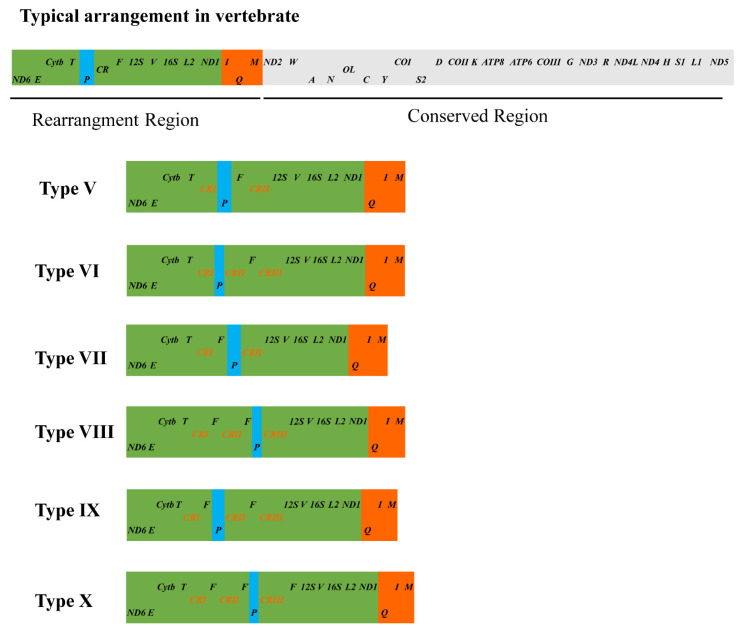
Type of the gene arrangements of the *Phrynocephalus* spp. mitogenomes. Genes encoded by the heavy and light strands are denoted at the top and bottom of each gene rectangle box, respectively. The sizes of the boxes do not reflect the actual gene length. OL, light strand replication origin. CR, control region (D-loop). Assignment of the rearrangement type number corresponds to that in Liu et al. [18].

In this study, the investigation of gene rearrangement events among 25 mitogenomes from 14 *Phrynocephalus* species revealed that there are six types of gene rearrangements (Figure 5, Table S6). Except for *P. frontalis* (GenBank accession no. MF039064), the rearrangement types of other 24 other mitogenomes are consistent with those of *Phrynocephalus* mitogenomes reported by Liu et al. [18]; while *P. frontalis* represents a novel rearrangement type (Type X). In the arrangement of mitochondrial genes, relative position variation of tRNA genes is more common than PCGs and rRNA genes [54]. Three types of tRNA gene position variation are observed in 25 mitogenomes of *Phrynocephalus*. The first is the gene rearrangement from IQM gene cluster (*tRNAIle*-*tRNAGln*-*tRNAMet*) to QIM, which supported the previous studies that the rearrangement of QIM gene cluster took place in an ancestral lineage of Agamidae + Chamaeleonidae [6,18,63]. The second is the change of *tRNA-Pro* location in *Phrynocephalus* mitogenomes, which exhibits extensive variations (Figure 5), including the translocation of the *tRNA-Pro* from the 5′ or 3′ end of *tRNA-Phe*, or the location of the *tRNA-Pro* between the two CRs. In the three *P. helioscopus* mitogenomes, *tRNA-Pro* of *P. helioscopus varius* II and *P. helioscopus cameranoi* is translocated to the upstream of *tRNA-Phe* and *tRNA-Pro* of *P. helioscopus varius* I is translocated to the downstream of *tRNA-Phe*. The third is the duplication of the *tRNA-Phe* occurring in *P. helioscopus* (except *P. helioscopus varius* I), *P. przewalskii*, *P. frontalis*, and *P. versicolor*. Interestingly, there are phenomena of intraspecific gene rearrangements in *P. helioscopus*, *P. erythrurus*, *P. vlangalii*, and *P. forsythii*. Intraspecific rearrangements of mitogenomes are rarely reported in reptiles, even in vertebrates [15], so it is necessary to further explore the mechanism of intraspecies rearrangements in *P. helioscopus*.

**Figure 6:**
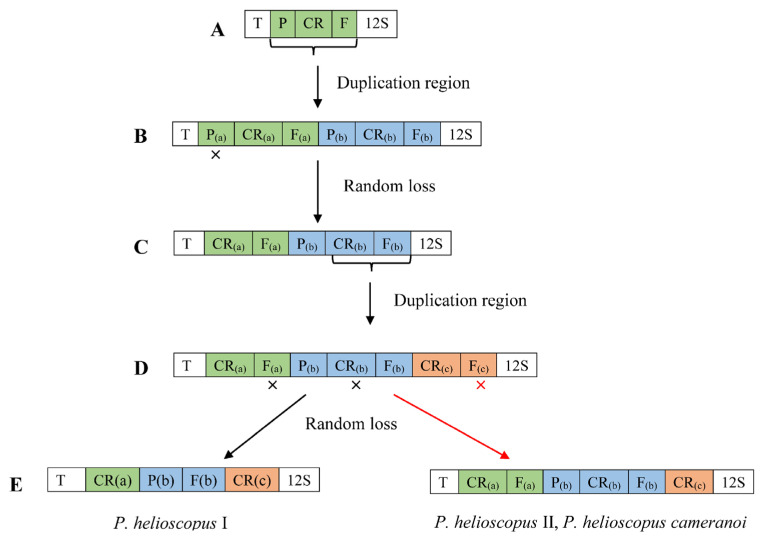
The hypothetical process of gene rearrangement in the model of tandem duplication/random loss: (**A**) the ancestral gene order in vertebrate; (**B**) the tandem duplication of P/CR/F; (**C**) the random loss of P(a); (**D**) the tandem duplication of CR(b)/F(b); (**E**) random loss of F(a) and CR(b), the formation of *P. helioscopus varius* I arrangement type; random loss of F(c), the formation of *P. helioscopus varius* II and *P. helioscopus cameranoi* arrangement type. “x” indicates the random loss of the genes; T, P, F, 12S and CR stand for Threonine, Proline, Phenylalanine, *12S rRNA*, control region, respectively.

At present, the most widely invoked model is tandem duplication of mitogenome genes followed by the random loss of copy (TDRL). Therefore, we hypothesize the gene rearrangement of mitogenomes of *P. helioscopus* based on the TDRL model. The steps of the TDRL are as follows (Figure 6). Firstly, the *tRNA-Pro*—CR—*tRNA-Phe* gene cluster was tandemly duplicated and generated two sets of the same gene cluster (*tRNA-Pro*_(a)_—CR_(a)_—*tRNA-Phe*_(a)_—*tRNA-Pro*_(b)_—CR_(b)_—*tRNA-Phe*_(b)_). Secondly, the *tRNA-Pro*_(a)_ gene was randomly eliminated, and the new order CR_(a)_—*tRNA-Phe*_(a)_—*tRNA-Pro*_(b)_—CR_(b)_—*tRNA-Phe*_(b)_ was generated. After that, the CR_(b)_—*tRNA-Phe*_(b)_ gene cluster duplicated again to form CR_(a)_—*tRNA-Phe*_(a)_—*tRNA-Pro*_(b)_—CR_(b)_—*tRNA-Phe*_(b)_—CR_(c)_—*tRNA-Phe*_(c)_ of the gene order. Finally, randomly eliminated *tRNA-Phe*_(a)_ and CR_(b)_ genes were generated the arrangement of *P. helioscopus varius* I (*tRNA-Thr*—CR_(a)_—*tRNA-Pro*_(b)_—*tRNA-Phe*_(b)_—CR_(c)_—*12S rRNA*), random eliminating *tRNA-Phe*_(c)_ gene, the arrangements of *P. helioscopus varius* II and *P. helioscopus cameranoi* were formed (*tRNA-Thr*—CR_(a)_—*tRNA-Phe*_(a)_—*tRNA-Pro*_(b)_—CR_(b)_—*tRNA-Phe*_(b)_—CR_(c)_—*12S rRNA*).

### 3.6. Phylogenetic Analysis

**Figure 7:**
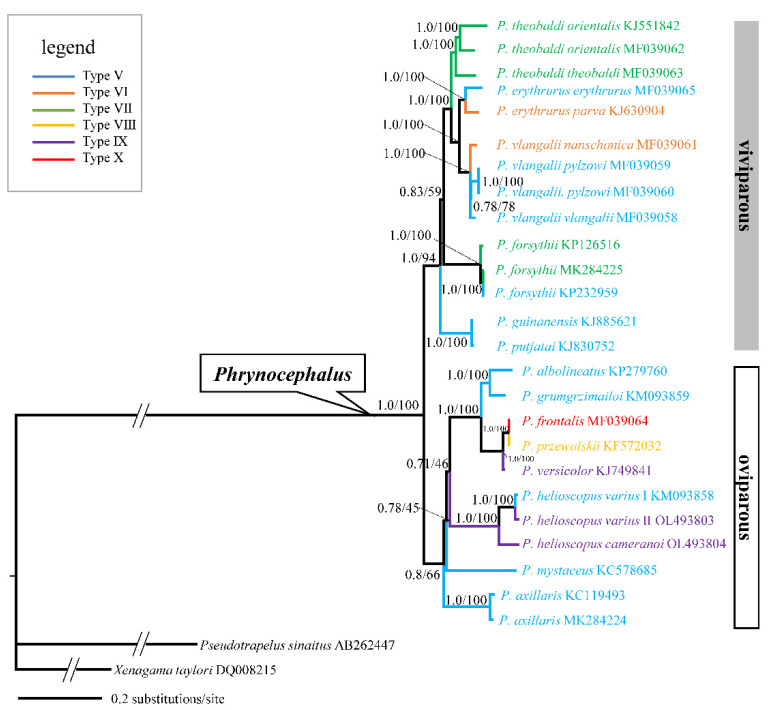
A majority-rule consensus tree inferred from Bayesian inference using MrBayes with the best models for each partition, based on the PCGs of 25 mitogenomes of *Phrynocephalus* and two outgroups. Node numbers show Bayesian posterior probabilities and ML ultrafast bootstrap values (UFBoot), respectively. Branch lengths represent means of the posterior distribution. GenBank accession numbers are given with species names, genus/group assignments are listed. Types V to X follow the types of mitochondrial gene arrangement patterns as designated in Liu et al. [18].

To further investigate the phylogenetic relationships of *P. helioscopus*, we constructed ML and BI trees based on the complete mitogenome of *P. helioscopus varius* II and *P. helioscopus cameranoi* along with 25 other Agamidae mitogenomes retrieved from GenBank. Both BI and ML approaches provided identical and well-supported tree topologies. Thus, only the BI tree is presented, which includes PP as well as UFBoot from ML (Figure 7). The phylogenetic tree strongly supports the monophyly of the viviparous group, which is consistent with previous studies [41,64,65,66,67]. The three individuals of *P. helioscopus* form a monophyletic group with strong support (PP = 1.0; UFBoot = 100), with the two individuals of *P. helioscopus varius* clustering together with strong support (PP = 1.0; UFBoot = 100). There is a relatively considerable *p*-distance between *P. helioscopu varius* and *P. helioscopu cameranoi* (7.1% and 6.9%). In contrast, *P. helioscopus varius* I and II only show a shallow divergence with *p*-distance of 1.5%. These results, in support of Solovyeya et al. [22], indicated that there is a deep genetic differentiation between *P. helioscopus cameranoi* distributed in the Ily River Valley and *P. helioscopus varius* distributed in the northern Junggar Basin.

A total of six types of mitochondrial gene rearrangements are observed in *Phrynocephalus*. As shown in Figure 7, multiple intraspecific gene rearrangements exist in *P. helioscopus* and other congeners including *P. erythrurus*, *P. vlangalii*, and *P. forsythii*. Overall, the occurrence of rearrangements may result from multiple independent tandem duplications and random loss events. However, the presence of extra tRNA gene is a rare event in the genus *Phrynocephalus*, even in reptiles. Phylogenetic analysis showed that species with tRNA duplication (*P. versicolor*, *P. przewalskii*, *P. helioscopus varius* II, and *P. helioscopus cameranoi*) are closely or far related oviparous species. Meanwhile, the existence of two types of gene rearrangements (Type V, IX) in *P. helioscopus* suggests that the duplication of tRNA may result from multiple independent structural dynamic events.

### 3.7. Divergence Time Estimation

**Figure 8:**
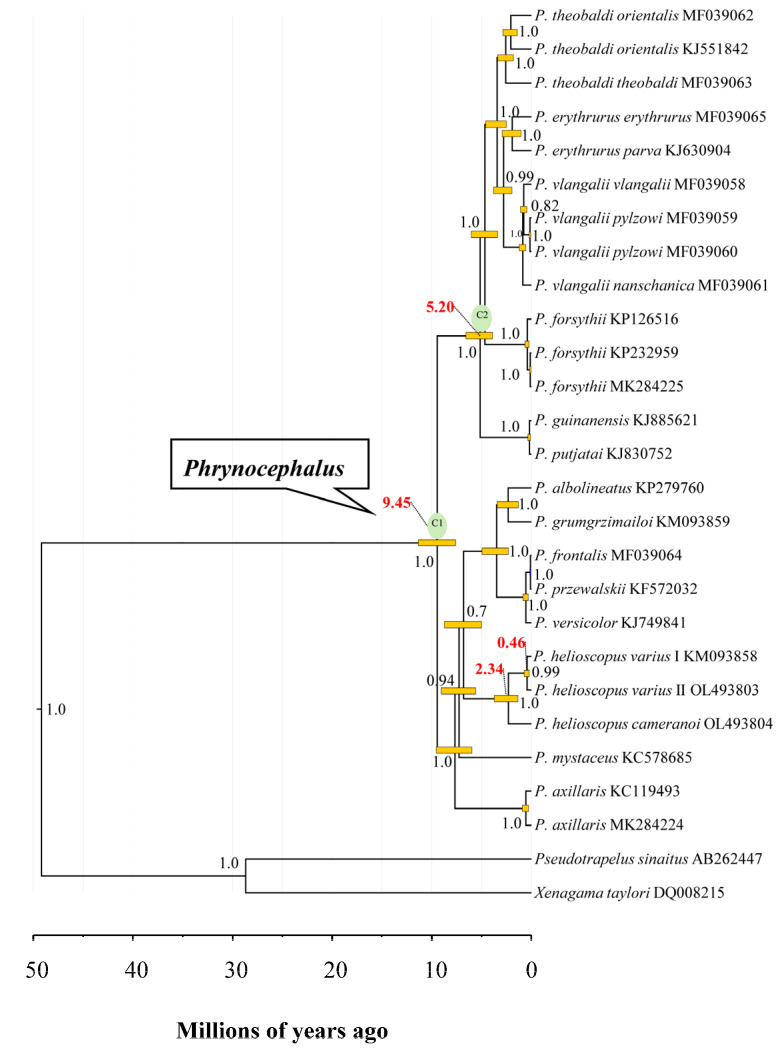
Timetree of *Phrynocephalus* spp. and two outgroup taxa estimated using BEAST based on 13 concatenated PCGs. Numbers on nodes (in black) are posterior probability values, while those in red highlight the mean divergence times in the *P. helioscopus* and *Phrynocephalus*. The yellow boxes indicate the 95% HPD of the node age in *Phrynocephalus*.

The most recent common ancestor of analyzed *Phrynocephalus* species was dated at approximately 9.45 Ma (95% HPD: 7.61–11.37 Ma) (Figure 8), which is consistent with previous studies [41,65,66]. The estimated splitting time between *P. helioscopus varius* I and *P. helioscopus varius* II was dated to approximately 0.46 Ma (95% HPD: 0.23–0.72 Ma), and *P. helioscopus cameranoi* diverged from *P. helioscopus varius* at approximately 2.34 Ma (95% HPD: 1.35–3.73 Ma). This implied that the differentiation of these two subspecies may be related to the rapid uplift event of the northern Tian Shan since 7–2.58 Ma [68,69]. The initiation of mitogenomic rearrangements in *P. helioscopus* possibly dates to about 2.34 Ma and divergence between the two types (Type V and IX) ranges from 0.23 to 0.72 Ma, suggesting that the duplication and fixation of these rearrangements can occur quite quickly. Combing interspecific rearrangement data, we may infer that post duplication, the alternative loss types could occur in 0.1–2 Ma.

## 4. Conclusions

In this study, by comparing the characteristics of mitogenomes of two subspecies *P. helioscopus cameranoi* and *P. helioscopus varius*, we observed the duplication of *tRNA-Phe* and the phenomenon of intraspecific rearrangements. We further speculate the rearrangement processes based on the TDRL model. Gene order and phylogenetic analyses support prevalent intraspecific gene rearrangement in *P. helioscopus* and other congener species including *P. erythrurus*, *P. vlangalii*, and *P. forsythii*. The split of the two subspecies was dated at approximately 2.34 Ma; this may be triggered by the rapid uplift event of the northern Tian Shan since 7–2.58 Ma. However, post duplication, the alternative loss types can occur in 0.72–0.23 Ma, suggesting that the duplication and fixation of these rearrangements can occur quite quickly. In addition, six types of mitochondrial gene rearrangements are observed in *Phrynocephalus*. Overall, the occurrence of rearrangements may result from multiple independent structural dynamic events. Our observation will provide clues to the investigations of intraspecific mitogenomic reorganization in *Phrynocephalus*. Further study is necessary to shed light on the evolutionary dynamics of the rearrangements in *Phryncephalus* by using intraspecific and population level investigations.

## Data Availability

Illumina raw reads are deposited at NCBI Sequence Read Archive (SRA) under accession no. SRR16925449 for *P. helioscopus varius* II in the BioProject PRJNA779875 and SRR16927567 for *P. helioscopus cameranoi* in the BioProject PRJNA779912. The mitochondrial genomes are deposited at GenBank with accession no. OL493803 for *P. helioscopus varius* II and OL493804 for *P. helioscopus cameranoi*.

## Supplementary Materials

The following supporting information can be downloaded at: https://www.mdpi.com/article/10.3390/genes13020203/s1, Figure S1: Putative secondary structures of the tRNAs identified in the mitogenomes of *P. helioscopus*, (a), *P. helioscopus varius* I; (b), *P. helioscopus varius* II; (c), *P. helioscopus cameranoi*. Table S1: *Phrynocephalus* and two outgroup species analyzed in this study with their GenBank accession numbers. Table S2: Data partitions, their estimated models of sequence evolution, and total number of characters of each partition used in phylogenetic analysis and molecular dating. Table S3: Characteristics of the three mitochondrial genomes of *P. helioscopus*. Table S4: Comparison of the composition and skew values of three mitogenomes for *P. helioscopus*. Table S5: Comparison of the codon usage in mitochondrial protein-coding genes for *P. helioscopus varius* I, *P. helioscopus varius* II and *P. helioscopus cameranoi*. Table S6: Twenty-five *Phrynocephalus* mitogenomes analyzed in gene rearrangement with their GenBank accession numbers.
