# Supplementary material for: Comparative Analysis of Mitochondrial Genomes in Two Subspecies of the Sunwatcher Toad-Headed Agama (Phrynocephalus helioscopus): Prevalent Intraspecific Gene Rearrangements in Phrynocephalus
Source: Genes (Basel). 2022 Jan 23;13(2):203. doi: 10.3390/genes13020203 (PMC8872181; doi:10.3390/genes13020203)
Supplement: Supplementary file 1 [file genes-13-00203-s001.zip › Table S1.pdf]

Table S1. *Phrynocephalus* and two outgroup species analyzed in this study with their GenBank accession numbers.

Taxon	Family	GenBank accession number	Size (bp)	Reference
Phrynocephalus albolineatus	Agamidae	KP279760	16,808	[S1]
Phrynocephalus axillaris	Agamidae	KC119493	17,937	[S2]
Phrynocephalus axillaris	Agamidae	MK284224	15,883	[S3]
Phrynocephalus erythrurus erythrurus	Agamidae	MF039065	16,456	[S4]
Phrynocephalus erythrurus parva	Agamidae	KJ630904	16,379	[S5]
Phrynocephalus forsythii	Agamidae	KP126516	17,542	[S6]
Phrynocephalus forsythii	Agamidae	KP232959	16,143	[S7]
Phrynocephalus forsythii	Agamidae	MK284225	16,060	[S8]
Phrynocephalus frontalis	Agamidae	MF039064	17,041	[S4]
Phrynocephalus grumgrzimailoi	Agamidae	KM093859	16,301	[S9]
Phrynocephalus guinanensis	Agamidae	KJ885621	16,279	[S10]
Phrynocephalus helioscopus cameranoi	Agamidae	OL493804	17,102	This study
Phrynocephalus helioscopus varius I	Agamidae	KM093858	16,249	[S11]
Phrynocephalus helioscopus varius II	Agamidae	OL493803	17,253	This study
Phrynocephalus mystaceus	Agamidae	KC578685	16,660	[S12]
Phrynocephalus przewalskii	Agamidae	KF572032	16,892	[S13]
Phrynocephalus putjatai	Agamidae	KJ830752	16,283	[S14]
Phrynocephalus theobaldi orientalis	Agamidae	KJ551842	16,608	[S15]
Phrynocephalus theobaldi orientalis	Agamidae	MF039062	17,129	[S4]
Phrynocephalus theobaldi theobaldi	Agamidae	MF039063	16,365	[S4]
Phrynocephalus versicolor	Agamidae	KJ749841	16,429	[S16]
Phrynocephalus vlangalii nanschanica	Agamidae	MF039061	15,251	[S4]
Phrynocephalus vlangalii pylzowi	Agamidae	MF039059	16,442	[S4]
Phrynocephalus vlangalii pylzowi	Agamidae	MF039060	16,725	[S4]
Phrynocephalus vlangalii vlangalii	Agamidae	MF039058	16,319	[S4]
Pseudotrapelus sinaitus	Agamidae	AB262447	16,560	Amer and Kumazawa (Unpublished data)
Xenagama taylori	Agamidae	DQ008215	16,220	[S17]

References in supporting materials

S1. Shao, M.; Ma, L.; Zhang, G.; Wang, Z. The complete mitochondrial genome of the toad-headed lizard, *Phrynocephalus albolineatus* (Reptilia, Squamata, Agamidae). *Mitochondrial DNA Part A* **2015**, *28*, 137–138.

S2. Li, J.; Guo, X.; Chen, D.; Wang, Y. The complete mitochondrial genome of the Yarkand toad-headed agama, *Phrynocephalus axillaris* (Reptilia, Squamata, Agamidae). *Mitochondrial DNA* **2013**, *24*, 234–236.

S3. Chen, D.; Guo, X. Next-generation sequencing yields a nearly complete mitochondrial genome of the Yarkand toad-headed agama (*Phrynocephalus axillaris*) from the Turpan Depression. *Mitochondrial DNA Part B* **2019**, 4, 1198–1199.

S4. Jin, Y.; Brown, R. Partition number, rate priors and unreliable divergence times in Bayesian phylogenetic dating. *Cladistics* **2017**, *34*, 568–573.

S5. Zhu, L.; Liao, P.; Tong, H.; Jin, Y. The complete mitochondrial genome of the subspecies, *Phrynocephalus erythrurus parva* (Reptilia, Squamata, Agamidae), a toad-headed lizard dwell at highest elevations of any reptile in the world. *Mitochondrial DNA Part A* **2014**, *27*, 703–704.

S6. Chen, D.; Zhou, T.; Guo, X. The complete mitochondrial genome of *Phrynocephalus forsythii* (Reptilia, Squamata, Agamidae), a toad-headed agama endemic to the Taklamakan Desert. *Mitochondrial DNA Part A* **2015**, 27, 4046–4048.

S7. Shao, M.; Ma, L.; Wang, Z. The complete mitochondrial genome of the toad-headed lizard, *Phrynocephalus forsythii* (Reptilia, Squamata, Agamidae). *Mitochondrial DNA Part A* **2016**, *27*, 3147–3148.

S8. Chen, D.; Li, J.; Guo, X. Next-generation sequencing yields a nearly complete mitochondrial genome of the Forsyth's toad-headed agama, *Phrynocephalus forsythii* (Reptilia, Squamata, Agamidae). *Mitochondrial DNA Part B* **2019**, 4, 817–819.

S9. Luo, S.; Liu, L.; Song, S. The complete mitochondrial genome of Grumgzimailo's toad-headed agama, *Phrynocephalus grumgrizimailoi* (Reptilia, Squamata, Agamidae). *Mitochondrial DNA Part A* **2014**, *27*, 1581–1582.

S10. Fu, C.; Chen, W.; Jin, Y. The complete mitochondrial genome of *Phrynocephalus guinanensis* (Reptilia, Squamata, Agamidae). *Mitochondrial DNA Part A* **2014**, *27*, 1103–1104.

S11. Li, D.; Guo, J.; Zhou, X.; Chang, C.; Zhang, S. The complete mitochondrial genome of *Phrynocephalus helioscopus* (Reptilia, Squamata, Agamidae). *Mitochondrial DNA Part A* **2014**, *27*, 1846–1847.

S12. Chen, D.; Guo, X.; Li, J. The complete mitochondrial genome of secret toad-headed agama, *Phrynocephalus mystaceus* (Reptilia, Squamata, Agamidae). *Mitochondrial DNA* **2014**, 25, 19–20.

S13. Li, D.; Song, S.; Chen, T.; Zhang, C.; Chang, C. Complete mitochondrial genome of the desert toad-headed agama, *Phrynocephalus przewalskii* (Reptilia, Squamata, Agamidae), a novel gene organization in vertebrate mtDNA. *Mitochondrial DNA* **2013**, 26, 696–697.

S14. Tong, H.; Jin, Y. The complete mitochondrial genome of an agama, *Phrynocephalus putjatia* (Reptilia, Squamata, Agamidae). *Mitochondrial DNA Part A* **2014**, 27, 1028–1029.

S15. Liao, P.; Jin, Y. The complete mitochondrial genome of the toad-headed lizard subspecies, *Phrynocephalus theobaldi orientalis* (Reptilia, Squamata, Agamidae). *Mitochondrial DNA Part A* **2014**, *27*, 559–560.

S16. Song, S.; Li, D.; Zhang, C.; Jiang, K.; Zhang, D.; Chang, C. The complete mitochondrial genome of the color changeable toad-headed agama, *Phrynocephalus versicolor* (Reptilia, Squamata, Agamidae). *Mitochondrial DNA* **2014**, *27*, 1121–1122.

S17. Macey, J.R.; Schulte, J.A.; Fong, J.J.; Das, I.; Papenfuss, T.J. The complete mitochondrial genome of an agamid lizard from the Afro-Asian subfamily agaminae and the phylogenetic position of *Bufoniceps* and *Xenagama*. *Mol. Phylogenet. Evol.* **2006**, 39, 881–886.
