# Supplementary material for: Comparative Analysis of Mitochondrial Genomes in Two Subspecies of the Sunwatcher Toad-Headed Agama (Phrynocephalus helioscopus): Prevalent Intraspecific Gene Rearrangements in Phrynocephalus
Source: Genes (Basel). 2022 Jan 23;13(2):203. doi: 10.3390/genes13020203 (PMC8872181; doi:10.3390/genes13020203)
Supplement: Supplementary file 1 [file genes-13-00203-s001.zip › Table S2.pdf]

Table S2. Data partitions, their estimated models of sequence evolution, and total number of characters of each partition used in phylogenetic analysis and molecular dating.

Partition No.	Model	Partition identity											Number of characters in partition
1	GTR+I+G	*ATP6-1st*	*ND4L-1st*	*ND2-1st*	*ND5-1st*	*ND4-1st*							1685
2	HKY+I+G	*ATP8-2nd*	*ND3-2nd*	*ATP6-2nd*	*ND4-2nd*	*ND4L-2nd*	*ND5-2nd*	*ND2-2nd*					1842
3	GTR+I+G	*COX1-3rd*	*ND4L-3rd*	*ND2-3rd*	*COX2-3rd*	*ATP6-3rd*	*Cytb-3rd*	*ND1-3rd*	*COX3-3rd*	*ND3-3rd*	*ND4-3rd*	*ND5-3rd*	3469
4	HKY+I	*ATP8-3rd*	*ATP8-1st*										100
5	TRN+I+G	*COX3-1st*	*COX1-1st*										769
6	TRN+I+G	*COX1-2nd*	*COX2-2nd*	*COX3-2nd*	*ND1-2nd*	*Cytb-2nd*							1677
7	TRN+G	*COX2-1st*	*ND3-1st*	*ND1-1st*	*Cytb-1st*								1015
8	HKY+G	*ND6-1st*	*ND6-2nd*										314
9	TRN+G	*ND6-3rd*											157
