# Supplementary material for: Comparative Analysis of Mitochondrial Genomes in Two Subspecies of the Sunwatcher Toad-Headed Agama (Phrynocephalus helioscopus): Prevalent Intraspecific Gene Rearrangements in Phrynocephalus
Source: Genes (Basel). 2022 Jan 23;13(2):203. doi: 10.3390/genes13020203 (PMC8872181; doi:10.3390/genes13020203)
Supplement: Supplementary file 1 [file genes-13-00203-s001.zip › Table S3.pdf]

Table S3. Characteristics of the three mitogenomes of P. helioscopus

(a) *P. helioscopus varius* I

Gene/Element	From	To	Anticodon	Length (bp)	Codon		Spacer (+)/Overlap	Strand
					Star	Sto		
tRNA-Phe	1	68	GAA	68				H
tRNA-Pro	72	87(incomplete)						L
CRII	88	421						
12S rRNA	422	1287		865				H
tRNA-Val	1285	1348	CAC	64			−3	H
16S rRNA	1349	2832		1484				H
tRNA-Leu (	2836	2909	TAA	74			+3	H
ND1	2907	3875		969	AT	TA	−3	H
tRNA-Gln	3872	3943	TTG	72			−4	L
tRNA-Ile	3952	4019	GAT	68			+8	H
tRNA-Met	4020	4082	CAT	63				H
ND2	4084	5118		1035	AT	AG	+1	H
tRNA-Trp	5118	5188	TCA	71			−1	H
tRNA-Ala	5189	5257	TGC	69				L
tRNA-Asn	5258	5329	GTT	72				L
tRNA-Cys	5346	5406	GCA	61			+16	L
tRNA-Tyr	5407	5473	GTA	67				L
COX1	5474	7021		1548	AT	AG		H
tRNA-	7013	7082	TGA	70			-9	L
tRNA-Asp	7086	7154	GTC	69			+3	H
COX2	7155	7839		685	AT	T		H
tRNA-Lys	7840	7902	TTT	63				H
ATP8	7903	8061		159	GT	TA		H
ATP6	8055	8736		682	AT	T	−7	H
COX3	8737	9520		784	AT	T		H
tRNA-Gly	9521	9585	TCC	65				H
ND3	9586	9925		340	AT	T		H
tRNA-Arg	9926	9993	TCG	68				H
ND4L	9994	10,284		291	GT	TA		H
ND4	10,27	11,642		1365	AT	TA	−7	H
tRNA-His	11,64	11,706	GTG	63			+1	H
tRNA-Ser(AGY)	11,70	11,767	GCT	61				H
tRNA-	11,76	11,838	TAG	71				H
ND5	11,84	13,609		1770	AT	TA	+1	H
ND6	13,59	14,079		483	GT	AG	−13	L
tRNA-Glu	14,08	14,148	TTC	69				L
Cytb	14,15	15,293		1141	AT	T	+4	H
tRNA-Thr	15,29	15,357	TGT	64				H
CRI	15,35	16,294		937				

(b) *P. helioscopus varous* II

Gene/Element	From	To	Anticodon	Length (bp)	Codon Start	Codon Stop	Spacer (+)/ Overlap (−)	Strand
tRNA-Phe	1	68	GAA	68				H
tRNA-Pro	72	133	TGG	62			+3	L
CRII	134	937		804				
tRNA-Phe	938	1005	GAA	68				H
CRIII	1006	1422		417				
12S rRNA	1423	2289		867				H
tRNA-Val	2287	2350	CAC	64			-3	H
16S rRNA	2351	3835		1485				H
tRNA-Leu (UUR	3839	3912	TAA	74			+3	H
ND1	3910	4878		969	AT	TAG	−3	H
tRNA-Gln	4875	4946	TTG	72			−4	L
tRNA-Ile	4955	5022	GAT	68			+8	H
tRNA-Met	5023	5085	CAT	63				H
ND2	5087	6121		1035	AT	AGA	+1	H
tRNA-Trp	6121	6191	TCA	71			−1	H
tRNA-Ala	6192	6260	TGC	69				L
tRNA-Asn	6261	6332	GTT	72				L
tRNA-Cys	6349	6409	GCA	61			+16	L
tRNA-Tyr	6410	6476	GTA	67				L
COX1	6477	8024		1548	AT	AGA		H
tRNA-Ser(UCN)	8016	8085	TGA	70			−9	L
tRNA-Asp	8089	8157	GTC	69			+3	H
COX2	8158	8842		685	AT	T		H
tRNA-Lys	8843	8905	TTT	63				H
ATP8	8906	9064		159	GT	TAA		H
ATP6	9058	9739		682	AT	T	−7	H
COX3	9740	10,523		784	AT	T		H
tRNA-Gly	10,52	10,588	TCC	65				H
ND3	10,58	10,928		340	ATT	T		H
tRNA-Arg	10,92	10,996	TCG	68				H
ND4L	10,99	11,287		291	GT	TAA		H
ND4	11,28	12,645		1365	AT	TAA	−7	H
tRNA-His	12,64	12,709	GTG	63			+1	H
tRNA-Ser(AGY)	12,71	12,770	GCT	61				H
tRNA-	12,77	12,841	TAG	71				H
ND5	12,84	14,612		1770	AT	TAG	+1	H
ND6	14,60	15,082		483	GT	AGG	−13	L
tRNA-Glu	15,08	15,151	TTC	69				L
Cytb	15,15	16,296		1141	AT	T	+4	H
tRNA-Thr	16,29	16,360	TGT	64				H
CRI	16,36	17,253		893				

(c) P. helioscopus cameranoi

Gene/Element	From	To	Anticodon	Length (bp)	Codon		Spacer (+)/Overlap (−)	Strand
					Start	Stop		
tRNA-Phe	1	66	GAA	66				H
tRNA-Pro	70	131	TGG	62			+3	L
CRII	132	937		806				
tRNA-Phe	938	1009	GAA	72				H
CRIII	1010	1281		272				
12S rRNA	1282	2149		868				H
tRNA-Val	2147	2210	TAC	64			−3	H
16S rRNA	2211	3694		1484				H
tRNA-Leu (	3698	3771	TAA	74			+3	H
ND1	3769	4737		969	ATA	TAG	−3	H
tRNA- Gln	4734	4805	TTG	72			−4	L
tRNA- Ile	4814	4882	GAT	69			+8	H
tRNA-Met	4883	4945	CAT	63				H
ND2	4947	5981		1035	ATT	AGA	+1	H
tRNA-Trp	5981	6050	TCA	70			−1	H
tRNA-Ala	6051	6119	TGC	69				L
tRNA-Asn	6120	6191	GTT	72				L
tRNA-Cys	6206	6268	GCA	63			+14	L
tRNA-Tyr	6269	6335	GTA	67				L
COX1	6336	7883		1548	ATG	AGA		H
tRNA-	7875	7944	TGA	70			−9	L
tRNA-Asp	7948	8017	GTC	70			+3	H
COX2	8018	8702		685	ATG	T		H
tRNA-Lys	8703	8765	TTT	63				H
ATP8	8766	8924		159	GTG	TAA		H
ATP6	8918	9599		682	ATG	T	−7	H
COX3	9600	10,383		784	ATG	T		H
tRNA-Gly	10,384	10,448	TCC	65				H
ND3	10,449	10,788		340	ATT	T		H
tRNA-Arg	10,789	10,856	TCG	68				H
ND4L	10,857	11,147		291	GTG	TAA		H
ND4	11,141	12,505		1365	ATG	TAA	−7	H
tRNA-His	12,507	12,569	GTG	63			+1	H
tRNA-	12,570	12,629	GCT	60				H
tRNA-	12,630	12,700	TAG	71				H
ND5	12,702	14,465		1764	ATG	TAG	+1	H
ND6	14,453	14,935		483	GTG	AGG	−13	L
tRNA-Glu	14,936	15,004	TTC	69				L
Cytb	15,009	16,149		1141	ATG	T	+4	H
tRNA-Thr	16,150	16,213	TGT	64				H
CRI	16,214	17,102		889				
