# Supplementary material for: Comparative Analysis of Mitochondrial Genomes in Two Subspecies of the Sunwatcher Toad-Headed Agama (Phrynocephalus helioscopus): Prevalent Intraspecific Gene Rearrangements in Phrynocephalus
Source: Genes (Basel). 2022 Jan 23;13(2):203. doi: 10.3390/genes13020203 (PMC8872181; doi:10.3390/genes13020203)
Supplement: Supplementary file 1 [file genes-13-00203-s001.zip › Table S4.pdf]

Table S4. Comparison of the composition and skew values of three mitogenomes for *P. helioscopus*.

Element	T			C			A			G			Length (bp)			A+T%			G+C%			AT-skew			GC-skew		
	I	*II*	*III*	*I*	*II*	*III*	*I*	*II*	*III*	*I*	*II*	*III*	*I*	*II*	*III*	*I*	*II*	*III*	*I*	*II*	*III*	*I*	*II*	*III*	*I*	*II*	*III*
Mitogenome	26.4	26.7	26.4	25.2	25.1	25.2	35.9	35.7	35.9	12.5	12.5	12.5	16,249	17,253	17,102	62.3	62.4	62.3	37.7	37.6	37.7	0.152	0.144	0.152	−0.337	−0.335	−0.337
PGCs	28.0	28.0	28.1	25.0	25.0	24.9	34.7	34.8	34.8	12.2	12.1	12.3	11,249	11,252	11,246	62.7	62.8	62.9	37.2	37.1	37.2	0.107	0.108	0.107	−0.344	−0.348	−0.339
tRNAs	25.3	28.8	29.0	23.0	19.1	18.8	36.0	32.6	32.4	15.8	19.6	19.9	1428	1542	1546	61.3	61.4	61.4	38.8	38.7	38.7	0.175	0.062	0.055	−0.186	0.013	0.028
rRNAs	22.3	22.1	21.5	23.1	23.3	23.7	37.4	37.6	37.8	17.2	17.0	17.1	2349	2352	2352	59.7	59.7	59.3	40.3	40.3	40.8	0.253	0.260	0.275	−0.146	−0.156	−0.162
CR Ⅰ	32.3	31.2	33.2	24.1	24.7	24.7	31.2	31.1	31.9	12.4	12.5	12.1	892	893	889	63.5	62.3	65.1	36.5	37.2	36.8	−0.328	−0.020	−0.017	−0.002	−0.342	−0.321
CR Ⅱ	36.2	31.4	34.5	20.1	24.6	24.6	34.1	31.2	31.8	9.6	12.8	12.3	334	804	806	70.3	56	66.3	29.7	37.4	36.9	−0.316	−0.041	−0.030	−0.004	−0.333	−0.354
CRIII	—	35.7	33.6	—	17.3	17.3	—	32.1	37.9	—	12.9	9.2	—	417	272	—	67.8	71.5	—	30.2	26.5	—	−0.053	0.060	—	−0.146	−0.306

Note: *I* stands for *P. helioscopus varius* I; *I*I stands for *P. helioscopus varius* II; *III* stands for *P. helioscopus cameranoi.*
