# Supplementary material for: Comparative Analysis of Mitochondrial Genomes in Two Subspecies of the Sunwatcher Toad-Headed Agama (Phrynocephalus helioscopus): Prevalent Intraspecific Gene Rearrangements in Phrynocephalus
Source: Genes (Basel). 2022 Jan 23;13(2):203. doi: 10.3390/genes13020203 (PMC8872181; doi:10.3390/genes13020203)
Supplement: Supplementary file 1 [file genes-13-00203-s001.zip › Table S5.pdf]

Table S5. Comparison of the codon usage in mitochondrial protein-coding genes for *P. helioscopus varius* I, *P. helioscopus varius* II and *P. helioscopus cameranoi*.

	P. helioscopus varius I		*P. helioscopus varius II*		*P. helioscopus cameranoi*	
Codon	Count	RSCU	Count	RSCU	Count	RSCU
UUU (F)	128	1.17	129	1.18	132	1.22
UUC (F)	90	0.83	89	0.82	84	0.78
UUA (L)	155	1.69	153	1.67	171	1.86
UUG (L)	35	0.38	36	0.39	25	0.27
CUU (L)	77	0.84	75	0.82	72	0.78
CUC (L)	41	0.45	45	0.49	49	0.53
CUA (L)	206	2.24	213	2.32	198	2.16
CUG (L)	37	0.4	28	0.31	36	0.39
AUU (I)	206	1.21	210	1.24	209	1.24
AUC (I)	135	0.79	128	0.76	127	0.76
AUA (M)	253	1.73	257	1.73	244	1.68
AUG (M)	40	0.27	40	0.27	46	0.32
GUU (V)	30	0.91	33	0.99	30	0.93
GUC (V)	21	0.64	20	0.6	17	0.53
GUA (V)	64	1.94	64	1.92	67	2.08
GUG (V)	17	0.52	16	0.48	15	0.47
UCU (S)	26	0.6	27	0.63	25	0.58
UCC (S)	46	1.07	45	1.05	43	0.99
UCA (S)	133	3.08	135	3.15	134	3.09
UCG (S)	9	0.21	7	0.16	9	0.21
CCU (P)	19	0.41	18	0.39	20	0.43
CCC (P)	33	0.71	35	0.76	29	0.63
CCA (P)	122	2.64	121	2.62	125	2.72
CCG (P)	11	0.24	11	0.24	10	0.22
ACU (T)	43	0.44	41	0.41	43	0.42
ACC (T)	122	1.24	127	1.28	130	1.28
ACA (T)	222	2.25	223	2.24	229	2.25
ACG (T)	7	0.07	7	0.07	5	0.05
GCU (A)	33	0.53	30	0.49	35	0.55
GCC (A)	103	1.66	104	1.7	105	1.66
GCA (A)	109	1.76	106	1.74	107	1.69
GCG (A)	3	0.05	4	0.07	6	0.09
UAU (Y)	42	0.76	40	0.72	51	0.91
UAC (Y)	69	1.24	71	1.28	61	1.09
UAA (*)	3	1.5	3	1.5	3	1.5
UAG (*)	2	1	2	1	2	1
CAU (H)	27	0.64	27	0.64	26	0.62
CAC (H)	58	1.36	58	1.36	58	1.38
CAA (Q)	109	1.95	108	1.93	105	1.89

CAG (Q)	3	0.05	4	0.07	6	0.11
AAU (N)	48	0.6	48	0.6	42	0.55
AAC (N)	111	1.4	113	1.4	112	1.45
AAA (K)	121	1.86	120	1.86	123	1.89
AAG (K)	9	0.14	9	0.14	7	0.11
GAU (D)	10	0.36	12	0.44	18	0.65
GAC (D)	45	1.64	43	1.56	37	1.35
GAA (E)	77	1.86	77	1.83	74	1.76
GAG (E)	6	0.14	7	0.17	10	0.24
UGU (C)	16	1.23	17	1.31	16	1.19
UGC (C)	10	0.77	9	0.69	11	0.81
UGA (W)	90	1.89	87	1.83	87	1.81
UGG (W)	5	0.11	8	0.17	9	0.19
CGU (R)	7	0.46	7	0.46	6	0.39
CGC (R)	5	0.33	5	0.33	7	0.46
CGA (R)	48	3.15	48	3.15	47	3.08
CGG (R)	1	0.07	1	0.07	1	0.07
AGU (S)	18	0.42	17	0.4	19	0.44
AGC (S)	27	0.63	26	0.61	30	0.69
AGA (*)	2	1	2	1	2	1
AGG (*)	1	0.5	1	0.5	1	0.5
GGU (G)	20	0.4	23	0.46	22	0.44
GGC (G)	55	1.09	54	1.07	48	0.96
GGA (G)	89	1.76	90	1.78	89	1.79
GGG (G)	38	0.75	35	0.69	40	0.8
