# Supplementary material for: Comparative Analysis of Mitochondrial Genomes in Two Subspecies of the Sunwatcher Toad-Headed Agama (Phrynocephalus helioscopus): Prevalent Intraspecific Gene Rearrangements in Phrynocephalus
Source: Genes (Basel). 2022 Jan 23;13(2):203. doi: 10.3390/genes13020203 (PMC8872181; doi:10.3390/genes13020203)
Supplement: Supplementary file 1 [file genes-13-00203-s001.zip › Table S6.pdf]

Table S6. Twenty-five *Phrynocephalus* mitogenomes analyzed in gene rearrangement with their GenBank accession numbers.

Taxon	GenBank accession number	Size (bp)	Rearrangement type
P. albolineatus	KP279760	16,808	Ⅴ
P. axillaris	KC119493	17,937	V
P. axillaris	MK284224	15,883	V
P. erythrurus erythrurus	MF039065	16,456	Ⅴ
P. erythrurus parva	KJ630904	16,379	Ⅵ
P. forsythii	KP232959	16,143	Ⅴ
P. forsythii	KP126516	17,542	Ⅶ
P. forsythii	MK284225	16,060	Ⅶ
P. frontalis	MF039064	17,041	Ⅹ
P. grumgrzimailoi	KM093859	16,301	V
P. guinanensis	KJ885621	16,279	V
P. helioscopus varius I	KM093858	16,249	V
P. helioscopus varius ＩＩ	OL493803	17,253	Ⅸ
P. helioscopus cameranoi	OL493804	17,102	Ⅸ
P. mystaceus	KC578685	16,660	V
P. przewalskii	KF572032	16,892	Ⅷ
P. putjatai	KJ830752	16,283	V
P. theobaldi orientalis	KJ551842	16,608	Ⅶ
P. theobaldi orientalis	MF039062	17,129	Ⅶ
P. theobaldi theobaldi	MF039063	16,365	Ⅶ
P. versicolor	KJ749841	16,429	Ⅸ
P. vlangalii vlangalii	MF039058	16,319	Ⅴ
P. vlangalii pylzowi	MF039059	16,442	Ⅴ
P. vlangalii pylzowi	MF039060	16,725	Ⅴ
P. vlangalii nanschanica	MF039061	15,251	Ⅵ

Note: the rearrangement type (V-IX) followed Liu et al. [18]; a novel rearrangement type was identified and numbered as X.
