# Supplementary figures and images for: Comparative Analysis of Mitochondrial Genomes in Two Subspecies of the Sunwatcher Toad-Headed Agama (Phrynocephalus helioscopus): Prevalent Intraspecific Gene Rearrangements in Phrynocephalus
Source: Genes (Basel). 2022 Jan 23;13(2):203. doi: 10.3390/genes13020203 (PMC8872181; doi:10.3390/genes13020203)

# (a) *P. helioscopus varius* I

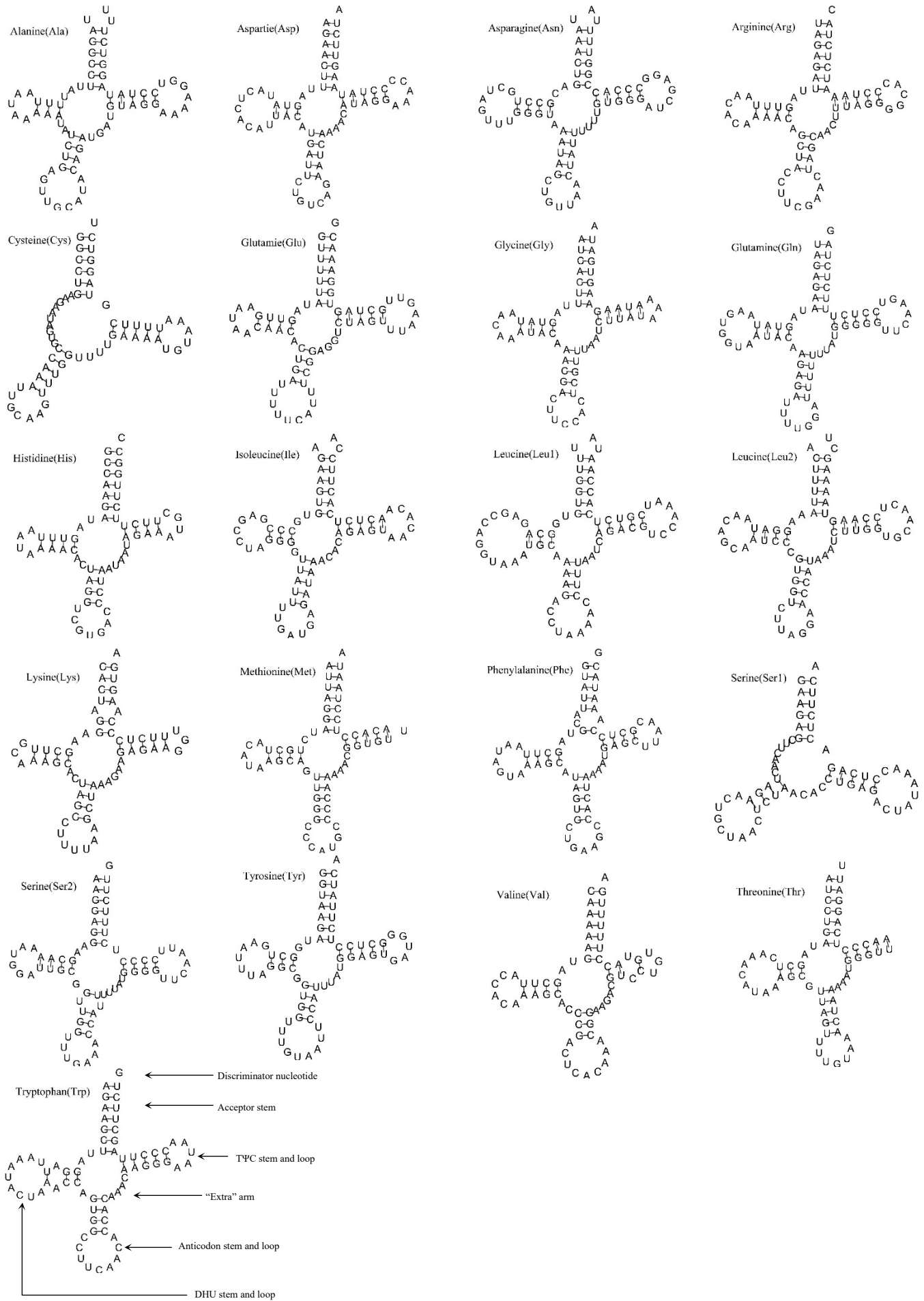

(b) *P. helioscopus varius* II

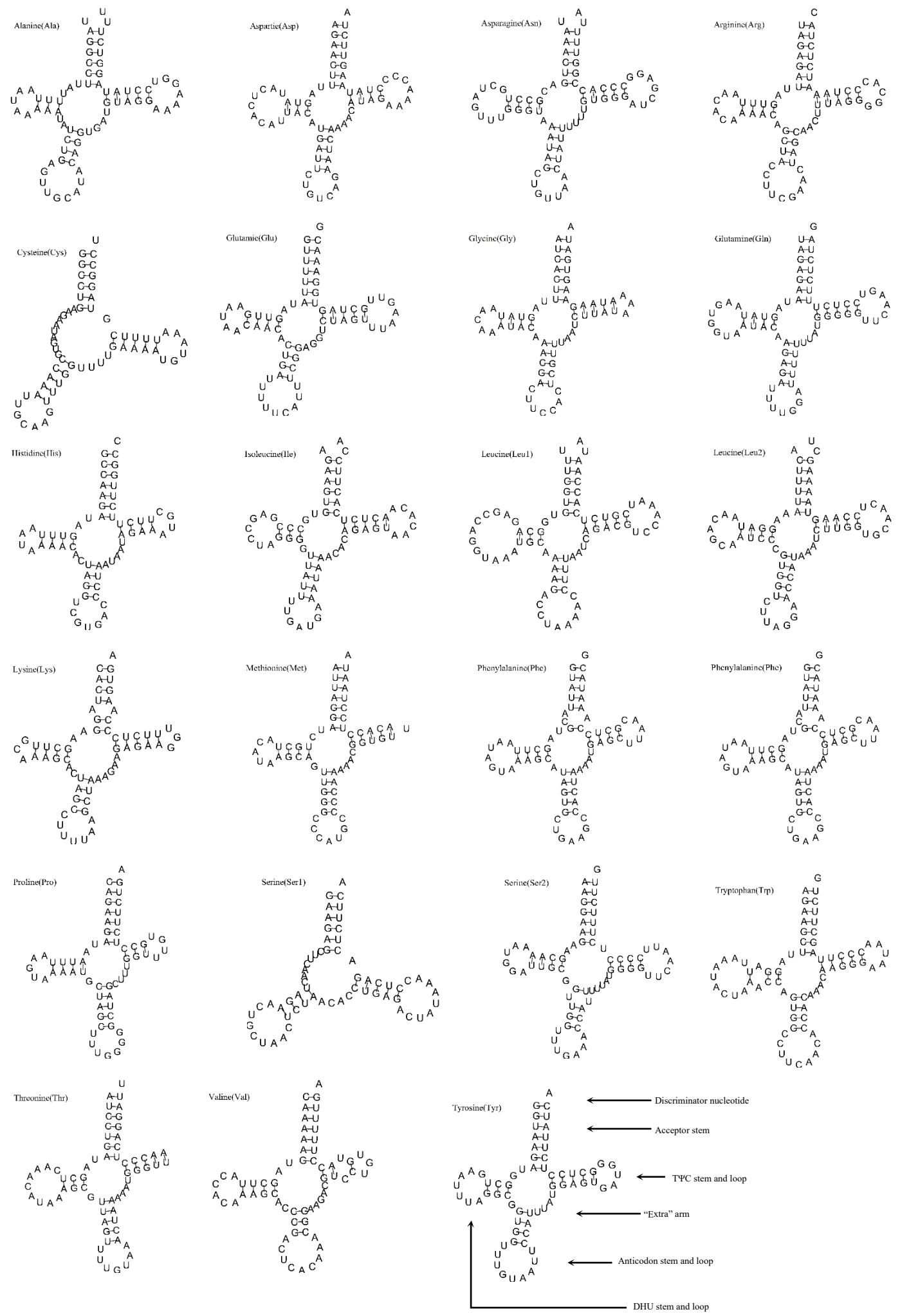

(c) *P. helioscopus cameranoi*

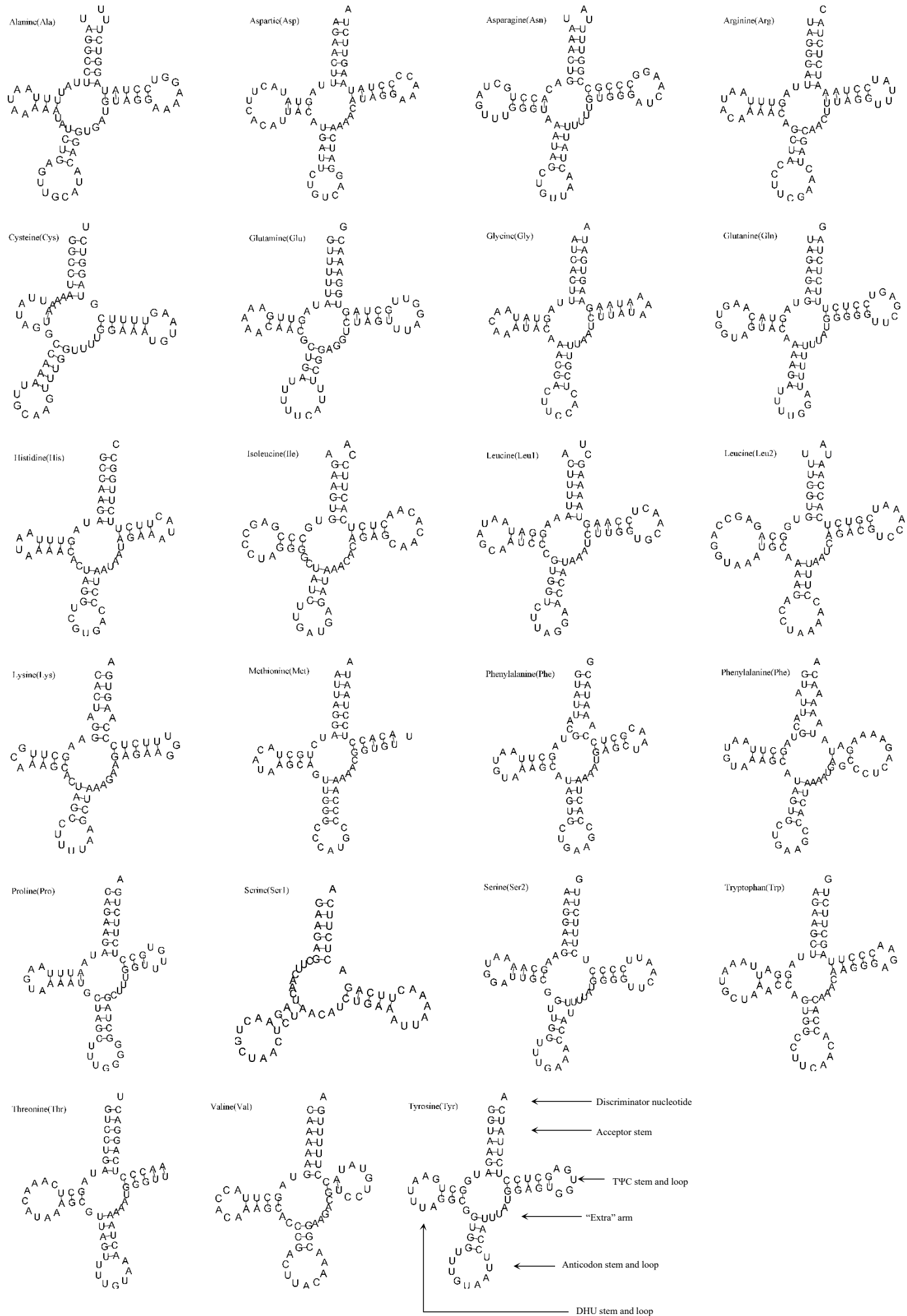

Supplement: Supplementary file 1 [file genes-13-00203-s001.zip › Figure S1.pdf]
